# Benzothiadiazinone-1,1-Dioxide Carbonic Anhydrase Inhibitors Suppress the Growth of Drug-Resistant *Mycobacterium tuberculosis* Strains

**DOI:** 10.3390/ijms25052584

**Published:** 2024-02-23

**Authors:** Silvia Bua, Alessandro Bonardi, Georgiana Ramona Mük, Alessio Nocentini, Paola Gratteri, Claudiu T. Supuran

**Affiliations:** 1 Research Institute of the University of Bucharest (ICUB), University of Bucharest, 050095 Bucharest, Romania; silviabua91@gmail.com; 2 Pharmaceutical and Nutraceutical Section, Laboratory of Molecular Modeling Cheminformatics & QSA, Neurofarba Department, University of Florence, Via U. Schiff 6, Sesto Fiorentino, 50019 Florence, Italy; paola.gratteri@unifi.it (P.G.); claudiu.supuran@unifi.it (C.T.S.); 3 Faculty of Biology, University of Bucharest, Splaiul Independenței 91-95, 050095 Bucharest, Romania; mukramonageorgiana@yahoo.com; 4 St. Stephen’s Pneumoftiziology Hospital, Șoseaua Ștefan cel Mare 11, 020122 Bucharest, Romania

**Keywords:** *Mycobacterium*, drug resistance, carbonic anhydrase, inhibitors, molecular modelling

## Abstract

2*H*-Benzo[e][1,2,4]thiadiazin-3(4*H*)-one 1,1-dioxide (BTD) based carbonic anhydrase (CA) inhibitors are here explored as new anti-mycobacterial agents. The chemical features of BTD derivatives meet the criteria for a potent inhibition of β-class CA isozymes. BTD derivatives show chemical features meeting the criteria for a potent inhibition of β-class CA isozymes. Specifically, three β-CAs (MtCA1, MtCA2, and MtCA3) were identified in *Mycobacterium tuberculosis* and their inhibition was shown to exert an antitubercular action. BTDs derivatives 2a-q effectively inhibited the mycobacterial CAs, especially MtCA2 and MtCA3, with K_i_ values up to a low nanomolar range (MtCA3, K_i_ = 15.1–2250 nM; MtCA2, K_i_ = 38.1–4480 nM) and with a significant selectivity ratio over the off-target human CAs I and II. A computational study was conducted to elucidate the compound structure-activity relationship. Importantly, the most potent MtCA inhibitors demonstrated efficacy in inhibiting the growth of *M. tuberculosis* strains resistant to both rifampicin and isoniazid—standard reference drugs for Tuberculosis treatment.

## 1. Introduction

*Mycobacterium tuberculosis*, also known as Koch bacillus, is the primary etiological agent responsible for the transmissible, chronic, and granulomatous infectious disease known as Tuberculosis (TB) [1]. In 2022 alone, this disease affected 10.6 million people and claimed the lives of 1.3 million individuals [1]. Among the infected population of all age groups, only 10% exhibit symptoms and are contagious [2]. Additionally, within this symptomatic group, only 50% survive with medical treatments [3].

TB primarily affects the lungs through the airborne transmission of bacteria when infected individuals cough, sneeze, or spit [4,5,6,7]. Upon entering the bloodstream, the bacteria can attack other organs and tissues, including the brain, kidneys, bones, spine, skin, peripheral lymph nodes, heart, pancreas, thyroid, spleen, eye, and skeletal muscles [8,9,10,11,12,13,14,15,16,17,18,19,20].

The common symptoms of TB vary depending on where in the body the infection becomes active and typically include a prolonged cough (sometimes with blood), chest pain, fatigue, weight loss, weakness, fever, and night sweats [1,4]. Several conditions, such as diabetes, a weakened immune system (e.g., HIV), malnutrition, and tobacco use, pose risk factors for Tuberculosis [21,22,23,24,25]. While the Bacille Calmette–Guérin (BCG) vaccine can be administered to infants or young children to prevent TB outside the lungs, it does not provide protection within the lungs [26]. Currently, the most effective vaccines are in phase I, II, and III of clinical trials [27,28,29,30]. Presently, the pharmacological treatment involves isoniazid and rifampicin for 4–6 months, representing the primary solution to combat TB infection [31,32].

Incorrect prescription or use of antibiotics, as well as premature therapy cessation, have led to the emergence of multidrug-resistant strains (MDR-TB) of *M. tuberculosis* against first-line drugs [33,34,35]. In such cases, second-line drugs like pyrazinamide, ethambutol, and streptomycin are employed [31,32]. However, anti-TB therapy shows serious side effects (e.g., hepatotoxicity) in almost 80% of the treated patients [36,37,38]. The ongoing research aims to develop more effective and safe medication to combat TB and MDR-TB [33,39].

Carbonic anhydrases (CAs, EC 4.2.1.1) constitute a superfamily of metalloenzymes that facilitate the reversible hydration of carbon dioxide into bicarbonate and a proton [40,41]. This reaction plays a crucial role in various physiological processes, including CO_2_ and HCO_3_^−^ transport, electrolytic secretion, biosynthetic reactions (such as gluconeogenesis, lipogenesis, and ureagenesis), tumorigenesis (in vertebrates), photosynthesis (in plants and cyanobacteria), pH regulation, virulence, growth, and the acclimatization of pathogens in specific niches [40,41].

Among the eight non-correlated gene families of CAs (α, β, γ, δ, η, ζ, θ, and ι) discovered to date, α, β-, γ-, and ι-class CAs have been identified in bacteria [40,41]. the activity of bacterial CAs is essential for supporting microbial central metabolism by maintaining the correct balance of CO_2_ and HCO_3_^−^, and these enzymes are promising and currently validated antibacterial targets [41,42,43,44,45]. Consequently, inhibiting carbonic anhydrase with inhibitors (CAIs) disrupts the growth of microbes and impedes virulence processes that render a pathogenic bacterium infectious to the host.

Notably, three β-Cas (MtCA1, MtCA2, and MtCA3) have been found in *M. tuberculosis*, and their selective inhibition could lead to antitubercular activity, exploiting an innovative antibacterial mechanism [46]. In this context, the inhibition of the α-class humans (h) CAs must be avoided to prevent the onset of side effects.

The identification of selective chemotypes for bacterial and, specifically, mycobacterial CA inhibition is one of the recently explored approaches for addressing the challenge of multidrug resistance, which is increasingly pushing us ever closer to the risk of a post-antibiotic era [47,48,49].

## 2. Results and Discussion

### 2.1. Design and Chemistry

β-CAs have rather narrow and flat active sites compared to the conical ones of the α-CAs, with many residues nearby the Zn^2+^ ion (bound to 2 cysteines and 1 histidine) capable of forming an extended H-bond network [50,51]. Therefore, zinc-binding chemotypes, similar to sulfonamides, endowed with a planar geometry and the capability to form a number of H-bonds, might be a weapon in the research of selective β-CA inhibitors. To date, several compounds have been identified as effective inhibitors of the β-CAs from *M. tuberculosis*, among which sulfonamides [52] and dithiocarbamates [53] demonstrated the most promising effects, with no significant selectivity of action reported to date against mycobacterial CAs over human isozymes.

**Scheme 1:**
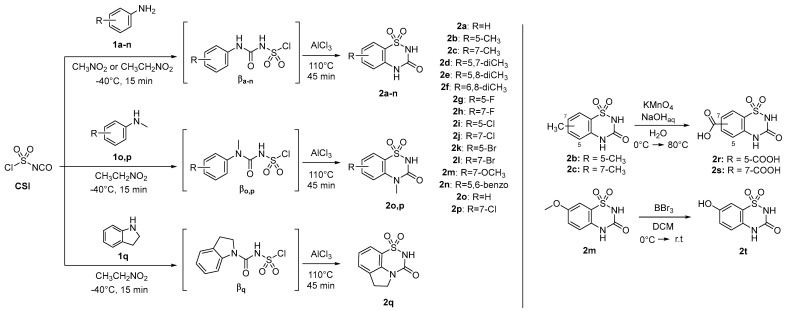
Synthesis of 2H-benzo[e][1,2,4]thiadiazin-3(4H)-one 1,1-dioxides **2a**-**2t** [54].

The 2H-benzo[e][1,2,4]thiadiazin-3(4H)-one 1,1-dioxides **2a**-**2t** (**BTDs**, Scheme 1) resulted from a preceding drug design for the development of antitumor CAIs, based on the hybridization of the scaffolds of two sweeteners, saccharin and acesulfame [54]. These compounds meet the aforementioned criteria for a potent binding to β-CAs and were never investigated earlier as inhibitors of bacterial CAs. Thus, they were re-prepared, according to the synthetic pathways of Scheme 1, to screen their β-CAs inhibitory action. As shown later in the article, potent and selective action against mycobacterial CAs was measured.

Briefly, the synthetic process involved the reaction of commercially available substituted anilines (**1a**-**1n**), N-methylanilines (**1o,1p**), and indoline (**1q**) with chlorosulfonyl isocyanate (CSI) at a temperature of −40 °C, utilizing nitroethane or nitromethane as a solvent. This reaction produced chlorosufamoyl urea intermediates **βa**-**q**. Subsequently, aluminum trichloride (AlCl_3_) was introduced, and the temperature was raised to 110 °C to facilitate a Friedel–Crafts-like core cyclization. The absence of a base and the maintenance of a very low temperature are crucial to preventing the aniline from reacting with the sulfamoyl portion of CSI. The choice between nitroethane and nitromethane is dictated by the need for a polar aprotic solvent that remains in a liquid state across a wide temperature range, from significantly below 0 °C to over 100 °C, without solidifying or boiling. Attempts to react markedly electron-poor anilines of the nitro-, sulfonamido-, or polyhalo-substituted types were unsuccessful due to their low reactivity in both reaction steps. Furthermore, the 5- and 7-methyl derivatives **2b** and **2c** underwent oxidation with potassium permanganate, yielding benzoic acids **2r** and **2s**, as depicted in Scheme 1. The 7-methoxy-**BTD** underwent demethylation upon treatment with 1.0 M BBr_3_ in DCM, resulting in the production of alcohol **2t** (Scheme 1).

### 2.2. Carbonic Anhydrase Inhibition Assay

**BTDs 2a**-**2t** were submitted to a stopped-flow CO_2_ hydrase kinetic assay [55] to evaluate the inhibitory profile against the mycobacterial β-CAs MtCA1, MtCA2, MtCA3 (Table 1), in comparison to the inhibitory effect on hCA I and II, which were selected due to their abundance in the human organism and thus off-target in this study.

As observed in Table 1, derivatives **2a**-**2q** effectively inhibited the investigated CA isozymes with K_i_ values ranging from 15.1 nM to greater than 10,000 nM. Notably, the most inhibited isozymes were MtCA3 (K_i_ = 15.1–2250 nM), MtCA2 (K_i_ = 38.1–4480 nM), hCA II (K_i_ = 140–6780 nM), MtCA1 (K_i_ = 556–>10,000 nM), and hCA I (K_i_ = 547–>10,000 nM), in descending order of potency.

Specifically, the incorporation of a methyl group (**2b**-**2d**) at positions 5 and/or 7 on the aromatic ring of the **BTD** scaffold variably enhances the inhibitory potency against MtCAs (**2b** > **2c** > **2d**), while diminishing the activity against hCAs. Conversely, a general weakening in inhibitory efficacy was noted for the 5,8- or 6,8-dimethyl substitutions (**2e**, **2f**) among all investigated isozymes.

The substitution with a fluorine atom at position 5 (**2g**) improves inhibition against MtCA3 and weakly against MtCA1, whereas the 7-fluoro derivative **2h** showed better K_i_ values only against MtCA2 than the unsubstituted compound **2a**.

The introduction of bulkier substituents (**2i**-**2m**, **2r**-**2t**), such as a chlorine/bromine atom or a methoxyl/carboxyl/hydroxyl group at positions 5 or 7, generally decreases inhibition against all investigated CAs compared to **2a**, except for the 5-Cl (**2i**) and 5-Br (**2k**) substitutions, which enhance K_i_ values against MtCA1, and the 7-OH inclusion (**2t**), which increases the MtCA-2 inhibition.

The 5,6-benzo condensation in the **BTD** scaffold (**2n**) enhances MtCA1 inhibition, while its inhibitory profile decreases against all other investigated CAs.

Derivative **2o** exhibits an increased inhibition profile against MtCA1 and 2, with a decreased K_i_ value against hCAs compared to **2a**, which was attributed to the presence of a methyl group on the N4 atom. Moreover, the simultaneous introduction of 7-Cl (**2p**) or a ethylene connection (**2q**) enhances activity exclusively against MtCA-1.

All compounds showed a more potent inhibition of hCA II than hCA I, except for compounds **2f** and **2t** (K_i_ values in the micromolar range). The selectivity index of inhibition of MtCAs over hCA II, as the main off-target and inhibited human isozyme, was calculated and is reported in Table 2. Most **BTDs** exhibited a preferential action against the hCA II compared to MtCA1 (SI < 1), with the exception of the N-substituted **2o**-**2q** and compounds **2s** and **2t** bearing hydrophilic substituents in position 7. In contrast, most **BTDs** demonstrated selectivity against MtCA2 and 3 over hCA II, ranging from 1.1 to 67. In detail, substituents appended at the 7 position of the scaffold promoted a higher selectivity of action compared to analog derivatives bearing a substitution in 5. This trend is even more marked for hCA II/MtCA3 (SI = 22.0–67.4) compared to hCA II/MtCA2 (SI = 11.8–51.8) selectivity. In fact, only a 5-substitution with a COOH moiety led to a SI hCA II/MtCA2 > 5. Instead, a greater number of 5-substituents lead to significant hCA II/MtCA2 SI (>5), such as a methyl, halogen, or carboxyl groups. It should also be noted that the N-methyl substitution (**2o**) improves the MtCA2 and 3 selectivity over hCA II.

### 2.3. In Silico Study

In silico studies were performed to thoroughly investigate the binding mode of **BTDs 2a**, **2c**, **2d**, **2h**, and **2t** within the active sites of the three β-Cas from *M. tuberculosis*. The aim was to unveil the relationship between the structural features and the inhibitory profile of the ligands. As the 3D-solved structure of MtCA3 is currently unavailable, we utilized the homology model (HM) previously built in our previous investigations [56,57].

**Figure 1:**
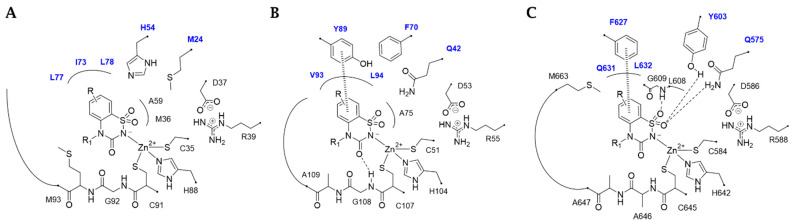
2D-Schematic representation of the binding mode of **BTDs** within the (**A**) MtCA1, (**B**) MtCA2, and (**C**) MtCA3 (HM) active site. The labels of amino acids from different chains are colored differently (Chain A: black, Chain B: blue).

In general, among the dimeric MtCAs active site, distinct structural variations contribute to a diverse binding mode of **BTDs**, explaining the different K_i_ values (Figure 1A–C).

Within the active sites of the three MtCAs, all docking solutions revealed the secondary sulfonamide bound to the zinc ion with the deprotonated nitrogen atom (SO_2_N^−^) adopting a tetrahedral geometry (Figure 1), consistent with the literature on the sulfonamides binding mode in the Cas active sites [40].

**Figure 2:**
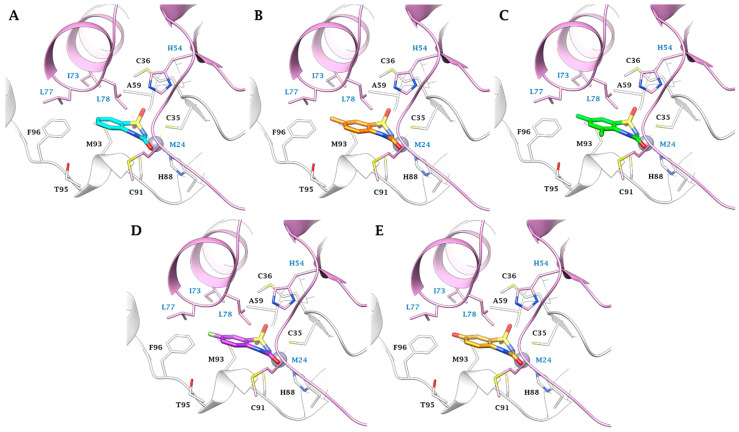
Predicted binding mode of ligands (**A**) **2a** (cyan), (**B**) **2c** (orange), (**C**) **2d** (green), (**D**) **2h** (purple) and (**E**) **2t** (gold) within the MtCA1 active site (pdb 1YLK). H-bonds are represented as black dashed lines, respectively. The labels of amino acids from different chains are colored differently (Chain A: black, Chain B: blue).

**Figure 3:**
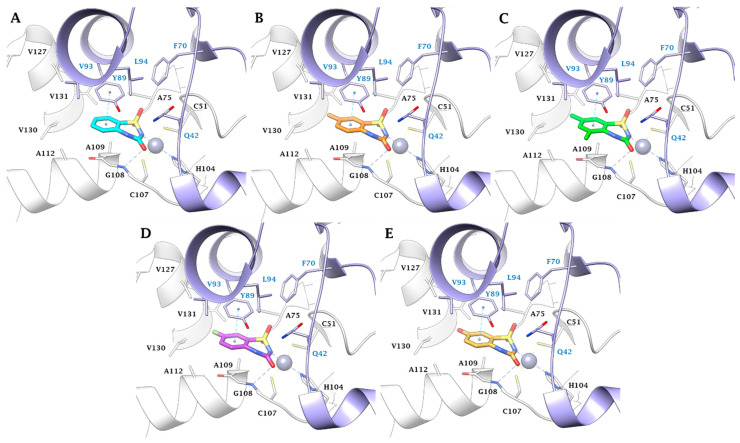
Predicted binding mode of ligands (**A**) **2a** (cyan), (**B**) **2c** (orange), (**C**) **2d** (green), (**D**) **2h** (purple), and (**E**) **2t** (gold) within the MtCA2 active site (pdb 2A5V). H-bonds and π-π stackings are represented as black and cyan dashed lines, respectively. The labels of amino acids from different chains are colored differently (Chain A: black, Chain B: blue).

**Figure 4:**
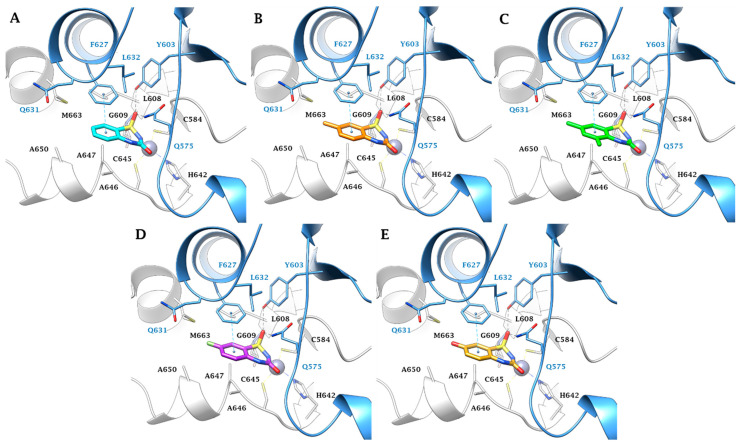
Predicted binding mode of ligands (**A**) **2a** (cyan), (**B**) **2c** (orange), (**C**) **2d** (green), (**D**) **2h** (purple), and (**E**) **2t** (gold) within the MtCA3 (HM) active site. H-bonds and π-π stackings are represented as black and cyan dashed lines, respectively. The labels of amino acids from different chains are colored differently (Chain A: black, Chain B: blue).

Furthermore, van der Waals (vdW) contacts with multiple hydrophobic residues play a significant role in reinforcing the metal binding coordination. These residues include M36(A), A59(A), G60(A), G92(A), M93(A), F96(A), M24(B), I73(B), L77(B), and L78(B) in MtCA1; A75(A), G75(A), G108(A), A109(A), A112(A), Q42(B), and Y89(B) in MtCA2; and Y603(A), L608(A), G609(A), A646(A), A647(A), M663(A), Q575(B), and F627(B) in MtCA3 (HM) (Figure 1, Figure 2, Figure 3 and Figure 4). In MtCA1, the orientation of the **BTD** scaffold is influenced by the steric hindrance of residues M93(A) and M24(B) (Figure 1A and Figure 2). Conversely, in MtCA2 and MtCA3 (HM), the crucial factors are π-π stacking interactions with Y89(B) and F627(B), respectively (Figure 1B,C, Figure 3 and Figure 4).

In the smaller active site of MtCA1, ligands **2a**, **2c**, **2d**, **2h**, and **2t** exhibit a binding mode characterized by hydrophobic contacts and shape complementarity, achieved through distinct substituents on the aromatic ring (Figure 2). Substituents like hydrogens (**2a**), methyl groups (**2c**, **2d**), or fluorine atoms (**2h**) are well accommodated (Figure 2A–D). Conversely, the tight cage lined by M93(A), T95(A), F96(A), M24(B), I73(B), L77(B), and L78(B) prevents the 8-substitution or the introduction of bulky groups (e.g., -Cl, -Br and -OCH3) or polar groups (e.g., -COOH and -OH (**2t**)) in position 5 or 7 (Figure 2E).

Within the MtCA2, the π-π stacking interaction between Y89(B) and the ligands aromatic ring, coupled with the M93(B)/A109(B) mutation (MtCA1/MtCA2), places the urea C=O of the **BTD** scaffold in H-bond distance with the backbone NH of G108(A) (Figure 3). The hydroxyl group of Y89(B) prevents the introduction of bulky groups in position 5 while allowing the 8-substitution with small groups. Additionally, the larger active site permits the hosting of larger or polar groups in position 7 of the aromatic ring (Figure 3B–E).

In the larger MtCA3 active site, the aromatic sidechain of F627(B) participates to a π-π stacking contact with the benzene ring of the ligands scaffold, allowing the formation of three H-bonds: one between the sulfonamide S=O and the backbone NH of G609(A), and two involving the other S=O group with the sidechains OH and NH_2_ of Y603(B) and Q575(B), respectively (Figure 4). The Y89(B)/F627(B) amino acid residues diversity between MtCA2/MtCA3 may thus be the explanation of the more effective inhibition profile of the 5-substituted ligands against the MtCA3 compared to the other MtCAs. Moreover, the roomier active site of MtCA3 better accommodates bulkier or polar substituent in position 7.

Despite the stabilizing effect of the I73(B)/Y89(B)/F627(B) mutation through π-π stacking within MtCA2 and MtCA3, it allows for the tolerance of substituents at position 8. In silico studies suggest that position 6 is the most favorable for functionalizing the 2H- **BTD** scaffold with larger pendants.

The ability of the **BTD** scaffold to establish strong interactions with MtCA3 > MtCA2 > MtCA1 elucidates the potent inhibition profile observed against MtCA3 and MtCA2 compared to MtCA1.

### 2.4. Tuberculostatic Activity Assay

Five of the most active compounds against MtCAs (**2a**, **2c, 2d**, **2h**, **2t**) were selected to evaluate their in vitro tuberculostatic activity towards three distinct strains with varying susceptibility profiles to the first-line clinical drugs rifampicin and isoniazid. This evaluation was conducted following a 28-day incubation period at 37 °C. The selected strains included one susceptible to both rifampicin and isoniazid, one susceptible to rifampicin and resistant to isoniazid, and one resistant to both rifampicin and isoniazid (Table 2).

In detail, while only compounds **2c**, **2d**, **2h** and the standard **AAZ** were active at the concentration of 1 µg/mL against the susceptible strain of *M. tuberuculosis* to rifampicin and isoniazid, all tested derivatives showed to inhibit the growth of mycobacteria at the increased concentration of 2 µg/mL. Except for **2t** at a concentration of 1 µg/mL, all compounds have completely inhibited the growth of the *M. tuberculosis* strains resistant to isoniazid and susceptible to rifampicin. Remarkably, all tested substances have inhibited the growth of the *M. tuberculosis* strain resistant to rifampicin and isoniazid at both concentrations (Table 3).

## 3. Material and Methods

### 3.1. Chemistry

Materials, methods, and synthetic procedures were previously reported [53].

### 3.2. Carbonic Anhydrase CO_2_ Hydration Catalytic/Inhibition Assay and Ki Determination

A stopped-flow instrument from Applied Photophysics was utilized to assess the inhibition of CA-catalyzed CO_2_ hydration activity [55]. Phenol red, present at a concentration of 0.2 mM, served as an indicator and operated at the absorbance peak of 557 nm. The assay employed 20 mM HEPES (pH 8.3) as a buffer and 20 mM Na_2_SO_4_ to maintain constant ionic strength. The investigation focused on the initial rates of the CA-catalyzed CO_2_ hydration reaction over a duration of 10–100 s. CO_2_ concentrations ranging from 1.7 to 17 mM were employed to determine kinetic parameters and inhibition constants. For each inhibitor, a minimum of six traces of the initial 5–10% of the reaction were utilized to determine the initial velocity. Uncatalyzed rates were determined in a similar manner and subtracted from the overall observed rates. Inhibitor stock solutions (0.1 mM) were initially prepared in distilled–deionized water, followed by subsequent dilutions up to 0.01 nM with the assay buffer. To allow for the formation of the E-I complex, inhibitor and enzyme solutions were preincubated together for 15 min at room temperature before the assay. The inhibition constants were derived through nonlinear least-squares methods using PRISM and the Cheng–Prusoff equation, as previously reported [51], representing the mean from at least three different determinations. Enzyme concentrations fell within the range of 6 to 19 nM, and all CA isoforms used were recombinant and obtained in-house, as detailed earlier [46].

### 3.3. In Silico Studies

The UniProt consortium was used to obtain the primary sequence of MtCA3. The homology modelling procedure used the 3D solved structure of β-CA from Synechocystis sp. PCC 6803 (PDB code 5SWC; resolution 1.45 Å) [58] as a template [56]. A large number of models were generated using the Prime module of Schrödinger [59] and the SwissModel platform [60]. These models were subjected to loop refinement and quality assessment procedures [56]. The highest scoring structure of MtCA3 and the crystal structures of CA I (PDB code 2NMX) [61], CA II (PDB code 3K34) [62], MtCA1 (PDB code 1YLK) [63], and MtCA2 (PDB code 2A5V) [64], downloaded from the Protein Data Bank (RCSB.org) [65], were prepared using the Protein Preparation module of the Maestro Schrödinger suite [60]. This involved the assignment of bond orders, the addition of hydrogens, the deletion of water molecules and the optimization of H-bond networks. The structures were then energy minimized to a Root Means Square Deviation (RMSD) of 0.30 using the Optimized Potential for Liquid Simulation (OPLS4) force field [66,67]. The 3D ligand structures were generated using Maestro [59]. The ionization states at pH 7.3 ± 1.0 were evaluated using Epik [59]. Energy minimization was performed using the conjugate gradient method in Macromodel [59] with a maximum iteration number of 2500 and a convergence criterion of 0.05 Kcal/mol/Å^2^. Docking studies were performed using the Glide program [59] in Standard Precision (SP) mode, with docking grids centered on the centroid of the complexed ligand. The resulting figures were generated using Chimera and Maestro [59,68].

### 3.4. Tuberculostatic Activity Assay

The tuberculostatic efficacy was evaluated against three different strains of *M. tuberculosis*, each exhibiting distinct susceptibility profiles—one susceptible to both rifampicin and isoniazid, another susceptible to rifampicin but resistant to isoniazid, and a third resistant to both rifampicin and isoniazid. The examined compounds were integrated into Lowenstein Jensen (LJ) solid medium at concentrations of 1 and 2 μg/mL. The tubes were positioned at a 45° angle in a thermostat set at 37 °C, ensuring the distribution of the tested substances within the culture medium. After 48 h, the inoculum was seeded onto the respective tubes, and the tubes were then incubated for 28 days. Simultaneously, the inoculum was also seeded onto a control tube that lacked the tested substances. To verify the sterility of the culture medium and the tested substances, unseeded samples were kept in the thermostat at 37 °C and observed for 28 days to confirm the absence of any growth or contamination. Following the 28-day incubation period, the colonies on media supplemented with varying concentrations of the tested substance were counted, along with colonies on the control tubes (one tube for each experiment). To ensure the validity of the results, it was crucial that numerous but countable colonies developed on the control tubes after 28 days of incubation. Result interpretation followed the absolute concentration method, where the growth of <30 colonies was deemed susceptible, while >30 colonies indicated resistance to the respective substance [69].

## 4. Conclusions

BTD-based CA inhibitors were investigated as novel anti-mycobacterial agents. The BTD framework aligns with the criteria identified for effectively inhibiting β-class CA isozymes. The three β-CAs (MtCA1, MtCA2, and MtCA3) encoded by *M. tubercolisis* play a pivotal role for the pathogen growth and virulence, and inhibiting them has demonstrated significant anti-tubercular effects. BTD derivatives **2a**-**q** have proven to be potent inhibitors of mycobacterial CAs, particularly MtCA2 and MtCA3, exhibiting K_i_ values in the low nanomolar range (MtCA3, K_i_ = 15.1–2254 nM; MtCA2, K_i_ = 38.1–4482 nM). Notably, these derivatives show a substantial selectivity ratio over human off-target CAs I and II. To better understand the structure-activity relationship of these BTD CA inhibitors, a comprehensive computational study was conducted. Crucially, the most effective MtCA inhibitors—**2a**, **2c**, **2d**, **2h**, **2t**, inhibited—the growth of *M. tuberculosis* strains resistant to both rifampicin and isoniazid, standard drugs in Tuberculosis treatment. This underscores the potential of BTD derivatives as promising candidates for the development of innovative anti-mycobacterial agents, emphasizing their ability to selectively target specific CA isozymes crucial for the survival of *M. tuberculosis*.

## Figures and Tables

**Table 1:** Inhibition data of β-CAs from *M. tuberculosis* (MtCA1, MtCA2, MtCA3) and human CA isozymes I and II with compounds **2a**-**2t** and acetazolamide (**AAZ**) as a standard drug, by a stopped-flow CO_2_ hydrase assay [55].

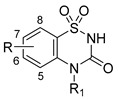							
			**K_i_ (nM) ^a^**				
**Cmpd**	**R**	**R_1_**	**MtCA1**	**MtCA2**	**MtCA3**	**hCA I ^b^**	**hCA II ^b^**
**2a**	H	H	1680 ± 93	68.6 ± 3.1	28.7 ± 2.1	866	150
**2b**	5-CH_3_	H	1460 ± 101	58.5 ± 1.7	21.3 ± 1.7	969	144
**2c**	7-CH_3_	H	1050 ± 65	41.3 ± 2.0	18.5 ± 1.6	1070	872
**2d**	5,7-diCH_3_	H	934 ± 78	38.1 ± 1.1	15.1 ± 0.9	1270	764
**2e**	5,8-diCH_3_	H	>10,000	2370 ± 167	1540 ± 91	2540	2190
**2f**	6,8-diCH_3_	H	>10,000	4480 ± 277	2250 ± 168	1920	2940
**2g**	5-F	H	1290 ± 96	62.9 ± 2.5	16.2 ± 1.1	547	140
**2h**	7-F	H	1830 ± 137	42.5 ± 1.6	24.3 ± 1.4	1290	534
**2i**	5-Cl	H	1500 ± 89	214 ± 13	44.3 ± 2.9	930	686
**2j**	7-Cl	H	2460 ± 174	67.1 ± 4.3	39.8 ± 2.2	1860	1140
**2k**	5-Br	H	993 ± 33	458 ± 25	82.4 ± 4.9	2320	527
**2l**	7-Br	H	2960 ± 213	200 ± 14	90.1 ± 6.7	4400	2360
**2m**	7-OCH_3_	H	2760 ± 166	152 ± 11	59.2 ± 4.2	3810	1920
**2n**	5,6-benzo	H	1040 ± 41	995 ± 35	120 ± 8	2070	484
**2o**	H	CH_3_	556 ± 37	39.6 ± 2.1	32.5 ± 2.4	3410	853
**2p**	7-Cl	CH_3_	1180 ± 75	135 ± 9	46.0 ± 3.3	>10,000	2340
**2q**	5-CH_2_-CH_2_-		883 ± 24	599 ± 31	76.2 ± 5.6	6860	1060
**2r**	5-COOH	H	3280 ± 204	105 ± 6	65.4 ± 3.9	5780	2450
**2s**	7-COOH	H	4870 ± 315	223 ± 18	101 ± 5	>10,000	6780
**2t**	7-OH	H	2390 ± 126	58.4 ± 4.1	45.6 ± 3.5	1890	3020
**AAZ**	-		480	9.8	104	250	12.5

^a^ Mean from three different assays, by a stopped-flow technique (errors were in the range of ±5–10% of the reported values); ^b^ K_i_ values from Ref. [54].

**Table 2:** Selective index (SI) for **BTD** compounds calculated as ratio between Ki against MtCAs and hCA II, as the main off-target isozyme.

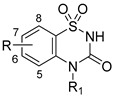					
			**Selectivity Index (SI)**		
**Cmpd**	**R**	**R_1_**	**hCA II/MtCA1**	**hCA II/MtCA2**	**hCA II/MtCA3**
**2a**	H	H	0.1	2.2	5.2
**2b**	5-CH_3_	H	0.1	2.5	6.8
**2c**	7-CH_3_	H	0.8	21.1	47.1
**2d**	5,7-diCH_3_	H	0.8	20.0	50.6
**2e**	5,8-diCH_3_	H	<0.2	0.9	1.4
**2f**	6,8-diCH_3_	H	<0.3	0.7	1.3
**2g**	5-F	H	0.1	2.2	8.6
**2h**	7-F	H	0.3	12.6	22.0
**2i**	5-Cl	H	0.5	3.2	15.5
**2j**	7-Cl	H	0.5	17.0	28.7
**2k**	5-Br	H	0.5	1.1	6.4
**2l**	7-Br	H	0.8	11.8	26.2
**2m**	7-OCH_3_	H	0.7	12.6	32.5
**2n**	5,6-benzo	H	0.5	0.5	4.0
**2o**	H	CH_3_	1.5	21.5	26.2
**2p**	7-Cl	CH_3_	2.0	17.3	50.8
**2q**	5-CH_2_-CH_2_-		1.2	1.8	13.9
**2r**	5-COOH	H	0.7	23.2	37.4
**2s**	7-COOH	H	1.4	30.4	67.4
**2t**	7-OH	H	1.3	51.8	66.3
**AAZ**	-		<0.1	1.3	0.1

**Table 3:** The number of *M. tuberculosis* colonies/standard inoculum volume (0.2 mL) recovered from the culture medium supplemented with different concentrations of the tested substances.

	Strain Susceptible to Rifampicin and Isoniazid		Strain Resistant to Isoniazid		Strain Resistant to Both Rifampicin and Isoniazid	
Cmpd	1 µg/mL	2 µg/mL	1 µg/mL	2 µg/mL	1 µg/mL	2 µg/mL
**2a**	30	0	0	0	0	0
**2c**	0	0	0	0	0	0
**2d**	0	0	0	0	0	0
**2h**	0	0	0	0	0	0
**2t**	30–100	0	30–100	0	0	0
**MTZ**	30–100	0	0	0	0	0
**AAZ**	0	0	0	0	0	0

## Data Availability

Data will be made available on request.
